# Supplementary material for: End Sequence Analysis Toolkit (ESAT) expands the extractable information from single-cell RNA-seq data
Source: Genome Res. 2016 Oct;26(10):1397–410. doi: 10.1101/gr.207902.116 (PMC5052061; doi:10.1101/gr.207902.116)
Supplement: Supplemental Material [file supp_gr.207902.116_Supp_Materials.tar.gz › ESAT-esat_v0.1/doc/html/functions_func_w.html]

ESAT: Class Members - Functions


ESAT


- Main Page
- Related Pages
- Packages
- Classes
- Files

- Class List
- Class Index
- Class Hierarchy
- Class Members

- All
- Functions
- Variables
- Enumerator

- a
- b
- c
- d
- e
- f
- g
- h
- i
- j
- k
- l
- m
- n
- o
- p
- q
- r
- s
- t
- u
- v
- w
- z


All Classes Namespaces Files Functions Variables Enumerator Pages

### - w -

- Wilcox2Sample()
  : broad.core.math.Wilcox2Sample
- WindowAllScore()
  : umms.core.model.score.WindowAllScore
- windowIterator()
  : umms.core.model.AlignmentModel
  , umms.core.model.JCSAlignmentModel
- WindowScoreIterator()
  : umms.core.model.score.WindowScoreIterator< T extends WindowScore >
- within()
  : broad.pda.datastructures.Alignments
- WrapAlignmentCountIterator()
  : umms.core.model.AlignmentModel.WrapAlignmentCountIterator
  , umms.core.model.JCSAlignmentModel.WrapAlignmentCountIterator
- WrappedIGVIterator()
  : broad.pda.seq.segmentation.WrappedIGVIterator
- WrapSamRecord()
  : umms.core.alignment.SingleEndAlignment.WrapSamRecord
- write()
  : broad.core.annotation.AnnotationReader< T extends GenomicAnnotation >
  , broad.core.datastructures.IntervalTree< V >
  , broad.core.datastructures.MatrixWithHeaders
  , broad.core.math.EmpiricalDistribution
  , broad.core.motif.PositionWeightColumn
  , broad.core.motif.PositionWeightMatrix
  , broad.core.motif.PositionWeightMatrixIO
  , broad.core.multiplealignment.FastaMultipleAlignmentIO
  , broad.core.multiplealignment.MAFAlignment
  , broad.core.multiplealignment.MAFIO
  , broad.core.multiplealignment.MultipleAlignment
  , broad.core.multiplealignment.MultipleAlignmentIO
  , broad.core.multiplealignment.PhylipInterleavedMultipleAlignmentIO
  , broad.core.multiplealignment.PhylipSequencialMultipleAlignmentIO
  , broad.core.sequence.FastaSequenceIO
  , broad.core.siphy.EvolutionaryModelParameters
  , broad.pda.annotation.GTFFileParser
  , broad.pda.datastructures.Alignments
  , broad.pda.datastructures.AlignmentsReader
  , broad.pda.datastructures.LocationAwareMatrix
  , broad.pda.geneexpression.clustering.HierarchicalClustering
  , broad.pda.geneexpression.PaGEJava
  , broad.pda.seq.fastq.FastqSequence
  , Jama.Matrix
  , umms.core.scripture.BuildScriptureCoordinateSpace
- writeAllAntisenseAlignmentsToBed()
  : umms.core.alignment.FeatureSequenceAlignment
- writeAllSenseAlignmentsToBed()
  : umms.core.alignment.FeatureSequenceAlignment
- writeAsText()
  : broad.core.sequence.FastaQualityIndex
- writeBarcodeCount()
  : broad.pda.seq.fastq.SplitFastqByIndex
- writeBED()
  : broad.pda.annotation.BEDFileParser
- writeBEDWithScores()
  : broad.pda.annotation.BEDFileParser
- writeBox()
  : broad.core.datastructures.MatrixWithHeaders
- writeChunks()
  : broad.pda.seq.fastq.FastqParser
- writeCLS()
  : broad.core.datastructures.MatrixWithHeaders
  , broad.pda.geneexpression.agilent.MakeCLSFromDescription
- writeClusters()
  : broad.pda.geneexpression.clustering.Kmeans
- writeClustersCentroids()
  : broad.pda.geneexpression.clustering.Kmeans
- writeColoumnNamesToFile()
  : broad.core.datastructures.MatrixWithHeaders
- writeConnections()
  : broad.pda.seq.segmentation.ContinuousDataAlignmentModel
- writeDistanceFromCentroids()
  : broad.pda.geneexpression.clustering.Kmeans
- writeDistanceFromInputCentroids()
  : broad.pda.geneexpression.clustering.Kmeans
- writeFasta()
  : broad.pda.rnai.ExtractSequence
  , broad.pda.seq.clip.FastaExtendPeaks
- writeFull()
  : broad.core.siphy.TreeScalerIO
- writeFullAsGenomeGraphUsingLogPval()
  : broad.core.siphy.TreeScalerIO
- writeFullBED()
  : broad.pda.annotation.BEDFileParser
- writeFullBed()
  : broad.pda.annotation.BEDFileParser
- writeFullBED()
  : broad.pda.annotation.BEDFileParser
  , broad.pda.seq.segmentation.ContinuousDataAlignmentModel
- writeGCT()
  : broad.core.datastructures.MatrixWithHeaders
  , broad.core.util.ParseGCTFile
- writeGCTAndCLS()
  : broad.core.datastructures.MatrixWithHeaders
- writeGCTWithHeaders()
  : broad.core.datastructures.MatrixWithHeaders
- writeGeneralStats()
  : broad.core.overlaputils.GeneSetIntersect
- writeGMT()
  : broad.core.datastructures.MatrixWithHeaders
  , broad.core.util.GMTParser
- writeGMTFile()
  : broad.core.util.ParseGCTFile
- writeGraph()
  : broad.pda.seq.graph.ChromosomeWithBubblesJGraphT
  , umms.core.scripture.OrientedChromosomeTranscriptGraph
- writeIndex()
  : broad.core.multiplealignment.MAFAlignment
- writeIntergenicGenome()
  : broad.pda.gene.transcriptsNullModel
- writeLeftJustifiedField()
  : broad.core.util.CLUtil
- writeNormalizedMatrix()
  : umms.esat.ESAT
  , umms.esat.ESATDistanceCalculator
- writeOverlap()
  : broad.core.overlaputils.GeneSetIntersect
- writeRecordsWithMinLength()
  : broad.core.sequence.FastaSequenceIO
- writeResult()
  : broad.pda.seq.protection.TwoSampleScanSkellamPeakCaller
- writeResults()
  : broad.pda.differentialExpression.RunDifferentialExpression
  , broad.pda.seq.protection.TwoSampleScanSkellamPeakCaller
- writeRightJustifiedField()
  : broad.core.util.CLUtil
- writeRowNamesToFile()
  : broad.core.datastructures.MatrixWithHeaders
- writeScanPvalsFragmentEndPileupsAllRegionsWig()
  : broad.pda.seq.clip.Pileup
- writeSeparateFiles()
  : broad.pda.countreads.FastqLibraryStats
- writeSequenceId()
  : broad.core.sequence.FastaSequenceIO
- writeSilhouetteRes()
  : broad.pda.geneexpression.clustering.Kmeans
- writeSingleSampleScanPeaks()
  : broad.pda.seq.protection.MultiSampleScanPeakCaller
- writeSizes()
  : broad.pda.feature.genome.DirectoryInstalledGenomeAssembly
- writeSortedBED()
  : broad.pda.annotation.BEDFileParser
- writeSortedBEDWithScores()
  : broad.pda.annotation.BEDFileParser
- writeTermsEnrichments()
  : broad.pda.gene.functionalAnnotation
- writeUNIPROBE()
  : broad.core.motif.PositionWeightMatrix
  , broad.core.motif.PositionWeightMatrixIO
- writeWigEachPosition()
  : broad.core.math.PermutationDistribution
- writeWindowsWithPvalue()
  : broad.core.math.PermutationDistribution


---

Generated on Fri Aug 15 2014 15:11:17 for ESAT by  

 1.8.7
